# Factors Affecting Oral Feeding Ability in Indonesian Preterm Infants

**DOI:** 10.3390/pediatric14020031

**Published:** 2022-05-13

**Authors:** Luh Karunia Wahyuni, Irawan Mangunatmadja, Risma Kerina Kaban, Elvie Zulka Kautzia Rachmawati, Melinda Harini, Budiati Laksmitasari, Boya Nugraha

**Affiliations:** 1 Department of Physical Medicine and Rehabilitation, Faculty of Medicine Universitas Indonesia—Dr. Cipto Mangunkusumo Hospital, Jakarta 10430, Indonesia; melindaharini@gmail.com (M.H.); blaksmitasari@gmail.com (B.L.); 2 Department of Child Health, Faculty of Medicine Universitas Indonesia—Dr. Cipto Mangunkusumo Hospital, Jakarta 10430, Indonesia; irawanma2802@gmail.com (I.M.); rismakk@yahoo.co.uk (R.K.K.); 3 Department of Ear, Nose and Throat Clinic (ENT), Faculty of Medicine Universitas Indonesia—Dr. Cipto Mangunkusumo Hospital, Jakarta 10430, Indonesia; zulka.elvie@gmail.com; 4 Department of Rehabilitation Medicine, Hannover Medical School, 30625 Hannover, Germany

**Keywords:** oral feeding ability, oral feeding readiness, preterm infants

## Abstract

Most preterm infants exhibit atypical and immature feeding skills. Even though preterm infants have fulfilled the oral feeding readiness criteria, they still do not have optimal oral feeding ability. This study aimed to determine various factors affecting oral feeding ability in Indonesian preterm infants who have fulfilled oral feeding readiness criteria but still have not been able to feed orally. A cross-sectional study included 120 preterm infants admitted to five tertiary hospitals in Jakarta, Indonesia. Participants were preterm infants born at 28–34 weeks gestational age who had fulfilled the oral feeding readiness as the inclusion criteria: (1) stable cardiorespiratory status, (2) have achieved full enteral feeding via orogastric tube (OGT) 120 mL/kg/day without vomiting or bloating, and (3) strong and rhythmic non-nutritive sucking (NNS) through objective measurement. Infants’ oral feeding ability and various factors that were assumed to affect oral feeding ability, including physiological flexion postural tone, physiological stability, rooting reflex, self-regulation, behavioral state, and level of morbidity were evaluated. Chi-square and multivariate analysis with Poisson regression were performed. Results indicated that postural tone, rooting reflex, physiological stability, self-regulation, behavioral state, and level of morbidity were significantly related to oral feeding ability in preterm infants. The most influencing factors were self-regulation with a prevalence ratio (PR) of 1.96 (1.16–3.34; CI 95%) and *p* = 0.012, followed by postural tone, high morbidity, and behavioral state (PR 1.91; 1.59; 1.56; CI 95%, respectively). In conclusion, despite meeting the oral feeding readiness criteria, most preterm infants were still not able to feed orally. There are other factors affecting oral feeding ability in Indonesian preterm infants.

## 1. Introduction

Indonesia is the fifth country with the highest number of preterm infants globally and is ranked seventh out of ten countries with the highest mortality [1]. Several studies have indicated that 40–70% of preterm infants exhibit atypical and immature feeding skills, require breathing apparatus, and experience delays in initiation of oral feeding [2]. Oral feeding ability reflects the infant’s adequacy to perform a series of complex processes that are very important for survival, and is one of the prerequisites for hospital discharge [3]. Several conditions that infants must achieve to be able to feed orally are: ability to engage and maintain physiological stability and behavioral state while feeding, ability to coordinate the suck-swallow-breathe process, ability to regulate and coordinate oral-motor functions, ability to perform airway protection in order to avoid prolonged apnea or fluid aspiration, and ability to maintain optimal postural tone [4].

Most preterm infants indicate problems in initiating sucking, having irregular, weak, inefficient sucking, and inability to coordinate suck-swallow-breathe, causing infants to get tired quickly during oral feeding [5]. These problems lead to difficulty transitioning from tube feeding to functional oral feeding. Prolonged gavage feeding affects the infant’s nutritional status, increases the length of stay and hospitalization cost, limits the emotional mother-child bonding, escalates the risk of maternal stress and re-admission to the Neonatal Intensive Care Unit (NICU), and may lead to long-term feeding difficulties [6]. Before preterm infants achieve their oral feeding ability, a non-oral feeding method is used to fulfill their nutritional needs. Two major enteral feeding routes for preterm infants are nasogastric and orogastric feeding. In the absence of high-quality evidence and clear guidelines that establish superiority between both methods, enteral feeding may vary in neonatal units and countries [7,8]. While most NICUs in Canada and Spain are using a nasogastric tube (NGT) as the primary option for enteral feeding in preterm infants, the application of an orogastric tube (OGT) is more common in Asian countries such as India and Indonesia [9,10,11]. Despite findings that it is more prone to displacement and vagal stimulation, OGT is preferred because it does not cause partial nasal obstruction, increased airway resistance, and work of breathing [7]. Decision-making of transition from orogastric to functional oral feeding requires evaluation of the infant’s oral feeding readiness. The state of oral feeding readiness should be based on an evaluation that captures the complexity of the oral feeding process and should be a universal language that can be understood by all medical teams [12]. 

Based on the researchers’ clinical practice experience, oral feeding readiness of a preterm infant is mainly determined by 32–34 weeks postmenstrual age (PMA), stable cardiorespiratory status, achieved full enteral feeding via orogastric tube, and strong non-nutritive sucking (NNS) using a gloved finger test. Even though preterm infants have fulfilled these oral feeding readiness criteria, they still do not have optimal oral feeding ability. Moreover, most infants continue to have feeding problems even after being discharged. It is assumed that other factors influence the oral feeding ability, and these factors have not been explored yet, particularly in Indonesia. This study aimed to determine various factors affecting oral feeding ability in Indonesian preterm infants who have fulfilled oral feeding readiness criteria but still have not been able to feed orally. 

## 2. Materials and Methods

This cross-sectional study was approved by the Research Ethics Committee of the Faculty of Medicine-Universitas Indonesia (protocol number: 21-03-0235), and parents consented for their infants to participate. Preterm infants born at 28 to 34 weeks gestational age admitted to five tertiary hospitals in Jakarta, Indonesia, were taken consecutively between August and November 2021. They were evaluated for the oral feeding readiness as the inclusion criteria: (1) stable cardiorespiratory status, (2) have achieved full enteral feeding via OGT 120 mL/kg/day without vomiting or bloating, and (3) strong and rhythmic non-nutritive sucking (NNS) evaluated by sucking mechanism evaluation system. Exclusion criteria were craniomaxillofacial malformations, neonatal asphyxia with a 5-min APGAR score less than 7, grade III and IV intraventricular hemorrhages, and receiving any respiratory support at the time of assessment.

The research team began screening for infants’ eligibility criteria approximately 30 min before their scheduled feed on the day of the visit. The sucking mechanism evaluation system is a non-invasive device for quantifying NNS in infants. This device consists of three parts: a data logger (GRAPHTEC midi LOGGER GL240, JTEKT Corporation, Kariya, Aichi Prefecture, Japan), an amplifier (JTEKT DC AMP AA6210, JTEKT Corporation, Kariya, Aichi Prefecture, Japan), and a pressure transducer (JTEKT PMS-5M2 50K, JTEKT Corporation, Kariya, Aichi Prefecture, Japan). A Preemie Care pacifier (Pigeon™, Pigeon Corporation, Tokyo, Japan) for the preterm infant was attached to the pressure transducer. The data logger allowed for real-time visualization and recording of the non-nutritive sucking pattern through an SD card. 

Once the device was set up and calibrated, an infant was swaddled in a physiological flexion, elevated to a 30-degree angle in bed, and the sides of the mouth were stroked with the pacifier to elicit a rooting response. The infant was ideally in a quiet-alert state; a pacifier was gently placed in the mouth, and NNS measurement was recorded for a 1-min period. Infants’ recorded sucking pattern data were exported and analyzed using Microsoft^®^ Excel for Mac version 16.16.27. Researchers were trained to identify strong and rhythmic NNS characteristics with the following criteria: 5–10 sucks per burst, 4–9 s between bursts, the amplitude of −16.7 to −87 mmHg, with a stable repetitive pattern. These criteria were adapted from previous studies quantifying NNS in preterm infants [13,14,15]. All data were saved as the participant’s ID number to avoid researcher bias during data analysis.

Infants who fulfilled the oral feeding readiness criteria above were then evaluated for their oral feeding ability for the first time. The infant was swaddled and supported in a semi-upright position. The oral feeding ability was evaluated using peristaltic plus nipple for low birth weight, in size SS (Pigeon™, Pigeon Corporation, Tokyo, Japan). Oral feeding ability was determined using the Oral Feeding Skill (OFS), an objective indicator of infants’ feeding ability that considers infants’ feeding skills and endurance, developed by Lau and Smith [16]. Oral feeding ability was achieved if infants could complete ≥30% of their prescribed feeding volume during the first 5 min, feeding rate ≥ 1.5 mL/min, and without any signs of aspiration. Infants were then classified into two groups based on their oral feeding ability: unable to feed orally and able to feed orally.

Infants in both groups were also evaluated for: (1) physiological flexion postural tone, (2) rooting reflex, (3) physiological stability while feeding, (4) self-regulation, (5) behavioral state, and (6) level of morbidity.

Physiological flexion postural tone was observed when the infant was in a supine position, at rest, and actively moving. Adequate physiological flexion postural tone was defined as: the infant’s neck was in the midline position, shoulders being protracted, hands pointing to face or mouth, with pelvic tilted posteriorly [17]. Rooting reflex was observed when the corners of the infant’s lips were stroked or touched. Rooting reflex was present if the infant turned their head and open their mouth to follow and root in the stroking direction [3]. Stability of the Cardiorespiratory System in Premature Infants (SCRIP) score was used to evaluate physiological stability while infant received oral feeding. Parameters assessed were heart rate, respiratory rate, and oxygen saturation. Three grades, from severe instability (0 points) to minor instability (1 points) to perfect stability (2 points), were chosen for each parameter. Physiological stability was fulfilled if the infant had 6 points, which describes that the infant has a regular heart rate of 120–160 bpm/minute, a regular respiratory rate 30–60 bpm/minute, and oxygen saturation continuously exceeding 90% [18]. Self-regulation was obtained through observation and was defined as the infant’s ability to modulate their system to engage in activities, interact with stimuli, maintain homeostasis, or withdraw from a stressful stimulus, and calm down when stress exceeded self-regulatory capacity [19]. Behavioral state was observed approximately 2 min before the infant’s feeding schedule in a swaddled position. Behavioral state assessment was adapted from Anderson Behavioral State Scale (ABSS); the alert state was described as an infant’s eyes being open and no movement to full-body movement observed [20]. Neonatal Medical Index (NMI) was used to evaluate the level of morbidity for each infant. The five levels in the NMI range from Level I, which describes a preterm infant relatively free of complicating problems, to Level V, which indicates that a preterm infant has the most significant medical complications [21].

Data analysis was performed through two stages of analysis: bivariate by using chi-square, and multivariate by using Poisson regression. Both analyses used STATA/SE 14.2 software. Prevalence ratio (PR) and *p* values were calculated for each factor. The level of significance is *p* < 0.05. 

## 3. Results

**Figure 1:**
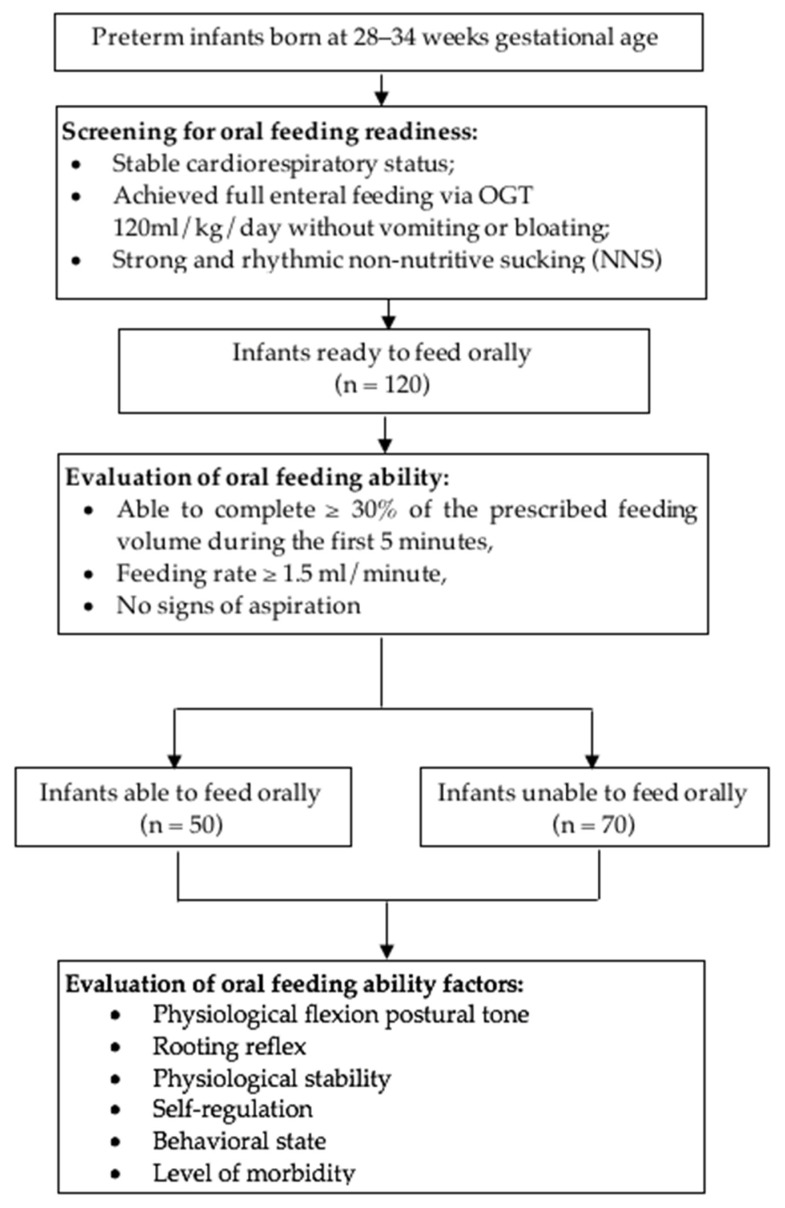
Flowchart of the study.

Out of the 120 preterm infants who were ready to feed orally, 50 were able to feed orally, and 70 were unable to feed orally, as portrayed in Figure 1. The data were analyzed from these preterm infants.

### 3.1. Characteristics of Study Population

Table 1 demonstrates the characteristics of the study population. There was a significant difference in infants’ sex (*p* = 0.002), with more female infants unable to feed orally (69%) compared to males (48.4%). Among 40 infants born at 28–31 weeks gestational age, only 9 (22.5%) were able to feed orally. Meanwhile, among 80 infants born at 32–34 weeks, 41 (51.2%) were able to feed orally (*p* = 0.003). There was no difference in Postmenstrual Age (PMA) between both groups (*p* = 0.051). Birth weight was significantly different between the two groups (*p* = 0.003). Birth weight was divided into three categories, with most of the infants being Low Birth Weight (LBW). This category consisted of 41 (53.2%) infants who were able to feed orally and 36 (46.8%) infants who were unable to feed orally. All comorbidities of preterm infants between the two groups were not significantly different. These comorbidities are bronchopulmonary dysplasia (BPD) (*p* = 0.130), intraventricular hemorrhage (IVH) (*p* = 0.269), necrotizing enterocolitis (NEC) (*p* = 0.948), apnea of prematurity (*p* = 0.396), and patent ductus arteriosus (PDA) (*p* = 0.290).

### 3.2. Factors Affecting Oral Feeding Ability 

Chi-square analysis of factors affecting oral feeding ability discovered that the postural tone, rooting reflex, and physiological stability had a very significant relationship with oral feeding ability in preterm infants (*p* < 0.001) (Table 2). According to the study results, preterm infants with an inadequate postural tone, no rooting reflex, and unstable physiological conditions while feeding were at twice the risk of inability to feed orally compared to infants who had adequate postural tone, rooting reflex, and stable physiological condition while feeding (PR 2.25; PR 2.09; PR 2.08; CI 95%). 

In addition, self-regulation, behavioral state, and high morbidity level were found to have a significant relationship with the oral feeding ability (*p* = 0.001; *p* = 0.026; and *p* = 0.011, respectively). Table 2 demonstrates that preterm infants who were ready to feed orally but unable to self-regulate had a 1.8 times greater risk of inability to feed orally than preterm infants who were able to self-regulate (PR 1.83; CI 95%). Preterm infants who were not alert during feeding had a 1.4 times greater risk of experiencing the inability to feed orally than preterm infants who were alert (PR 1.45; CI 95%). Furthermore, high-morbidity preterm infants have a 1.6 times greater risk of experiencing an inability to feed orally than preterm infants with low morbidity (PR 1.68; CI 95%).

### 3.3. Multivariate Analysis of Factors Affecting Oral Feeding Ability 

Multivariate analysis with Poisson regression as demonstrated in Table 3 indicated that self-regulation was the main factor affecting the preterm infants’ oral feeding ability, with PR 1.96 (1.1–3.3; CI 95%) and *p* = 0.012. Physiological flexion postural tone was the second most important factor with PR 1.91 (1.4–2.6; CI 95%) and *p* < 0.001, followed by high morbidity and behavioral state (PR 1.59; PR 1.56; CI 95%, respectively). Meanwhile, moderate morbidity was not significant in affecting preterm infants’ oral feeding ability (PR 1.15; CI 95%) with *p* > 0.05.

## 4. Discussion

The criteria of oral feeding readiness in this study were based on the criteria of oral feeding readiness used in the five hospitals where the study was conducted. Unlike the usual subjective measurement of NNS in those hospitals, the evaluation of NNS in this study was based on an objective measurement using the sucking mechanism evaluation system device. The characteristics of objective NNS measurement used in this study were representative of the NNS ability in infants aged 32–36 weeks PMA [13,14,15].

The study results indicated that most preterm infants who had fulfilled the criteria for oral feeding readiness were not able to feed orally. This study proved that NNS ability does not always represent nutritive sucking (NS) ability as a prerequisite of oral feeding. The findings in this study reflect the basic physiology of NNS, which is different from NS. Non-nutritive sucking occurs in the absence of swallowed liquid, and no pharyngeal phase of swallowing occurs. In NNS, the sucking and breathing processes run independently [22]. It proves that NNS alone is not a good indicator of oral feeding readiness because it cannot indicate the coordination between sucking, swallowing, and breathing. Several other factors must be considered, including the infant’s gestational or postmenstrual age, physiological stability, behavioral state, rooting reflex, postural tone, self-regulation, and level of morbidity.

### 4.1. Factors Affecting Oral Feeding Ability

**Physiological flexion postural tone.** The bivariate analysis found that physiological flexion postural tone was the most influential in oral feeding ability. Infants with inadequate postural tone had twice the risk of experiencing the inability to feed orally (PR 2.25; *p* < 0.001). This finding supports the physiological basis of the importance of the oral structure alignment for feeding, which cannot be separated from head and trunk stability [23,24,25]. Physiologically, overall muscle tone strongly influences oral-motor control. To achieve optimal oral feeding, the muscle tone of the infants must be in balance. The muscle tone should be sufficient to allow movement but not so high that it interferes with the flexibility of movement. The typical posture of full-term newborns is physiological flexed. The physiological flexion posture facilitates body alignment and movement. The posture is characterized by strong limb flexor muscle tone and the increased stretch reflex. The neck and trunk are flexed, the shoulders are protracted, the ribs are in a parallel and horizontal position, the upper chest is flat and narrow, the space between the ribs and pelvis is narrow, the pelvis is tilted posteriorly, and the trunk extensor muscles are in elongation [3,26,27,28,29].

**Rooting reflex.** Infants demonstrate the ability to pay attention to the feeding process through readiness to participate and be interested in sucking. The Shaker theory suggests that an infant seeking a nipple or rooting reflex early in the feeding indicates a neurologically mature readiness for oral feeding [3]. The intact rooting reflex of the baby will indicate adequate sucking ability because a rooting reflex often accompanies the sucking reflex. The rooting reflex is generated by light touch to the cardinal point reflex (the point on the cardinal edge). Our findings indicate that the absence of a rooting reflex has significant relationship to the inability to feed orally (*p* < 0.001). Infants who did not exhibit the rooting reflex were at twice the risk of experiencing inability to feed orally (PR 2.09; CI 95%). 

**Physiological stability**. Infants who can regulate physiological stability will be able to regulate their breathing patterns during oral feeding without increasing breathing effort. Adequate oxygenation will improve the behavioral state and produce sufficient energy for effective and efficient oral feeding. Inadequate oxygenation contributes to fatigue, leading to short feeding times and reduced caloric intake [30]. The results of this study indicate that physiological stability had a significant relationship to the oral feeding ability (*p* < 0.001), and it was found that preterm infants who were physiologically unstable while feeding had a twice greater risk of experiencing inability to feed orally (PR 2.08; CI 95%). Similar results were found in a previous study, which indicated that the ability to feed orally in preterm infants is determined by organizing oral-motor functions, swallowing and breathing coordination, and maintaining physiological stability. Physiological stability is characterized by sufficient oxygen saturation in hemoglobin (SaO2). Adequate oxygen saturation plays an essential role in the oral feeding ability of preterm infants. The initial value of oxygen saturation is the starting point for the stability of the autonomic system. Adequate oxygen saturation also affects the stability of respiratory function and reduces the risk of bradycardia and hypoxemia while feeding [3]. 

**Self-regulation**. Self-regulation had a significant effect (*p* = 0.001) on the oral feeding ability. Preterm infants who cannot perform self-regulation have a 1.8 times greater risk of experiencing an inability to feed orally (PR 1.83; CI 95%). Self-regulation is the culmination of behavioral development that contributes to the infant’s ability to regulate body systems to maintain balance when faced with external and internal stimuli. Infants with good self-regulation will develop coping skills and the ability to deal with stimuli that disrupt self-balance. Meanwhile, the infants will develop an approach if the stimulus provides a sense of comfort [31,32,33].

**Behavioral state**. Behavioral state determines the infant’s ability to interact or interest in carrying out functional activities, such as regulating the oral feeding process. The behavioral state of maturity allows the baby to wake up when hungry, maintain consciousness during the feeding process, transition to deep sleep, and rest for the next feeding. Stable infants will demonstrate sleep behavior that is deep, clear, regular, and comfortable. They cry loudly but effectively and are able to calm down. When awake, the infants will be focused, have bright eyes, be expressive, and smile. When hungry, infants move their hands to their mouth, suck on a pacifier, suck a finger or fidget, whine, and cry [34,35,36,37]. The study result found that an alert behavioral state affected the oral feeding ability (*p* = 0.026), and infants who were not alert during feeding had a 1.4 times greater risk of being unable to feed orally (PR 1.45; CI 95%). This finding supports the previous study by Griffith et al. in 2017 [34], stating that behavioral states (alert, sleeping, and crying) in preterm infants before feeding can significantly predict the efficiency of oral feeding. The efficiency of oral feeding increases in the alert or crying state, while the efficiency decreases if the infant is mainly asleep before feeding. Therefore, oral feeding while the infant is in the sleep state should be avoided. Another study concluded that behavioral state affects the ability of preterm infants to feed orally. Preterm infants in an alert state demonstrated better oral feeding ability [35]. 

**Level of Morbidity**. In this study, the level of morbidity was determined based on the Neonatal Medical Index (NMI). Level of morbidity was divided into three categories: low morbidity (NMI grades I–II), moderate morbidity (NMI grade III), and high morbidity (NMI grades IV–V). The study results indicate that the level of morbidity had a significant effect on the preterm infants’ oral feeding ability. Infants with high morbidity were 1.6 times more likely to experience an inability to feed orally than infants with a low morbidity (PR 1.68; CI 95%). The infants with moderate morbidity were 1.1 times more likely to experience the inability to feed orally compared to infants with low morbidity (PR 1.14; CI 95%). Research conducted by Liu et al. in 2010 [38] also reported a similar result, which indicates that the more significant the morbidity experienced by preterm infants, the more likely there will be various comorbid conditions affecting the ability to feed orally. These could be muscle hypertonus, difficulty in handling, poor self-regulation, less than optimal movement quality, and a high risk of stress.

**Multivariate analysis** indicated self-regulation as the main factor influencing the ability to feed orally with PR 1.96 (1.78–2.93; CI 95%) and *p* = 0.012, followed by postural tone, high morbidity, and behavioral state. This finding reflects the widely accepted Als’s Synactive Theory of Infant Development [39], which provides a framework for understanding preterm infants’ behavior. This theory describes five subsystems: autonomic, motor, states, attention/interaction, and self-regulatory as the presence and success of the infant’s effort to achieve and maintain balance of the other four subsystems. Recommendations of some strategies to enhance self-regulation by encouraging stability of the motor system, including positioning to promote flexion, hand-to-mouth, and grasping and tucking motions [40]. Furthermore, a study by Grenier and Bigsby in 2003 [40] also supports that several positioning techniques such as prone nested/un-nested and the side-lying nested position in preterm infants may cause less stress and make them able to self-regulate than when placed in other positions.

Supporting Oral Feeding in Fragile Infants (SOFFI) is a method of cue-based bottle-feeding developed by Ross and Philbin in 2011 [41], guiding the development of each infant into a competent feeder without a direct focus on volume intake. By providing a bedside algorithm, this method defines an efficient feeding as one where the infant self-regulates and remains stable throughout the feeding, and the caregiver supports the infant by monitoring cues and supporting with the utilization of strategies. Although the SOFFI method and this study were both based on the Als’s Synactive Theory, they are different in purpose, and several factors were not included in the SOFFI method, such as physiological flexion postural tone, rooting reflex, and level of morbidity, which were proven to affect oral feeding ability in preterm infants.

One of the highlights of this study was that 70 out of 120 preterm infants who had fulfilled the oral feeding readiness criteria were not able to feed orally. This finding proves that the oral feeding readiness criteria used in this study (stable cardiorespiratory status, full enteral feeding, and strong and rhythmic NNS) are still not comprehensive enough. Since these criteria were also used in five hospitals where the study was conducted, this represents the current condition of preterm infant management in Indonesia. Thus, a comprehensive protocol integrating self-regulation, physiological flexion postural tone, behavioral state, and level of morbidity with the current oral feeding readiness criteria is needed to provide better feeding management for preterm infants in Indonesia.

### 4.2. Clinical Implications

This study contributes to the clinical application of the teams involved in preterm infant management, particularly in Indonesia, that [1] in the process of transitioning the fulfillment of nutritional needs from an orogastric tube to an oral route, it is better to determine the status of the infant’s feeding readiness before trying to feed orally because infants who appear to be demonstrating signs of feeding readiness are not necessarily correlated with feeding success, [2] an objective evaluation of non-nutritive sucking needs to be added to the determination of oral feeding readiness, [3] the evaluation of infant’s self-regulation should be the first step in preparing preterm infants to be able to feed orally, and [4] there is a need for a comprehensive protocol to assess oral feeding readiness in preterm infants.

### 4.3. Limitations

Several potential limitations must be acknowledged for this study. First, infants may already have NNS experience prior to the start of the study, potentially altering their suck patterning. Therefore, future studies should control for previous pacifier use. In addition, as a cross-sectional study, this study could not determine a causal relationship between the level of morbidity, physiological stability, behavioral state, rooting reflex, self-regulation, and postural tone on the oral feeding ability of preterm infants. Future study is needed to know the causal relationship with a longitudinal study design conducted in multi-center throughout the country. It is also necessary to develop validated diagnostic tools to objectively assess several risk factors for the oral feeding ability in preterm infants. Further study is also needed to compare the correlation between subjective clinical examination and the objective sucking mechanism evaluation system to evaluate non-nutritive sucking ability, knowing that this objective measurement device was not always available in the hospitals.

## 5. Conclusions

The study indicates that despite meeting the oral feeding readiness criteria, most preterm infants were still not be able to feed orally. There are other factors affecting oral feeding ability in Indonesian preterm infants. The main factor influencing oral feeding ability was self-regulation, followed by postural tone, high morbidity, and behavioral state.

## Figures and Tables

**Table 1:** Characteristics of Study Population.

Characteristics	Unable to Feed Orally (n = 70)	Able to Feed Orally (n = 50)	*p* Value
**Sex, n (%)**			
Male	30 (48.4)	32 (51.6)	0.002 *
Female	40 (69.0)	18 (31.0)	
**Gestational age, weeks, n (%)**			
28–31	31 (77.5)	9 (22.5)	0.003 *
32–24	39 (48.8)	41 (51.2)	
**PMA, weeks, n (%)**			
32–34	38 (50.7)	37 (49,3)	0.051
35–36	25 (75.8)	8 (24.2)	
37–42	7 (58.3)	5 (41.7)	
**Birth weight, grams, n (%)**			
ELBW (<1000)	4 (80.0)	1 (20.0)	0.003 *
VLBW (1000–1499)	30 (78.9)	8 (21.1)	
LBW (1500–2499)	36 (46.8)	41 (53.2)	
**Comorbidities, n (%)**			
BPD	6 (85.7)	1 (14.3)	0.130
IVH	12 (70.6)	5 (29.4)	0.269
NEC	4 (57.1)	3 (42.9)	0.948
Apnea of Prematurity	2 (40.0)	3 (60.0)	0.396
PDA	10 (71.4)	4 (28.6)	0.290

Abbreviations: PMA = postmenstrual age; ELBW = extremely low birthweight; VLBW = very low birthweight; LBW = low birthweight; BPD = bronchopulmonary dysplasia; IVH = intraventricular hemorrhage; NEC = necrotizing enterocolitis; PDA = patent ductus arteriosus. Notes: Statistical significance was defined as * *p* < 0.05.

**Table 2:** Bivariate analysis on factors affecting oral feeding ability.

Factors	Unable to Feed Orally (n = 70)	Able to Feed Orally (n = 50)	PR (CI 95%)	*p* Value
**Postural tone, n (%)**				
Inadequate	33 (97.1)	1 (2.9)	2.25 (1.75–2.89)	<0.001 **
Adequate	37 (43)	49 (57)		
**Rooting reflex, n (%)**				
No	28 (96.6)	1 (3.4)	2.09 (1.65–2.63)	<0.001 **
Yes	42 (46.2)	49 (53.8)		
**Physiological stability, n (%)**				
Unstable	24 (100)	0 (0)	2.08 (1.69–2.57)	<0.001 **
Stable	46 (47.9)	50 (52.1)		
**Self-regulation, n (%)**				
Unable	55 (68.8)	25 (31.2)	1.83 (1.19–2.80)	0.001 *
Able	15 (37.5)	25 (62.5)		
**Behavioral state, n (%)**				
Not alert	49 (66.2)	25 (33.8)	1.45 (1.01–2.06)	0.026 *
Alert	21 (45.7)	25 (54.3)		
**Morbidity (NMI), n (%)**				
High (NMI IV-V)	18 (81.8)	4 (18.2)	1.68 (1.13–2.49) ^a^	0.011 *
Moderate (NMI III)	35 (55.5)	28 (44.5)	1.14 (0.76–1.71) ^b^	0.519
Low (NMI I-II) ^#^	17 (48.5)	18 (51.5)	**Reff**	

Notes: NMI = Neonatal Medical Index. ^#^ Low morbidity (NMI I–II) was used as a reference; * *p* < 0.05; ** *p* < 0.001. ^a^ PR of high morbidity to low morbidity. ^b^ PR of moderate morbidity to low morbidity.

**Table 3:** Multivariate analysis of factors affecting oral feeding ability.

Factors	PR (CI 95%)	*p* Value
Self-regulation	1.969 (1.161–3.340)	0.012 *
Postural tone	1.913 (1.400–2.614)	<0.001 **
High morbidity (NMI IV–V)	1.590 (1.312–2.234)	0.007 *
Behavioral State	1.568 (1.155–2.131)	0.004 *
Moderate morbidity (NMI III)	1.158 (0.826–1.622)	0.394

* *p* < 0.05; ** *p* < 0.001.

## Data Availability

The data presented in this study are available on request from the corresponding author. The data are not publicly available due to privacy issues.
